# Cement Burn

**Published:** 2015-02-24

**Authors:** Nigel Yong Boon Ng, Anas Abdullah, Stephen M. Milner

**Affiliations:** Johns Hopkins Burn Center, The Johns Hopkins University School of Medicine, Baltimore, Md

**Keywords:** cement, burn, alkaline, chemical injury, treatment

**Figure:**
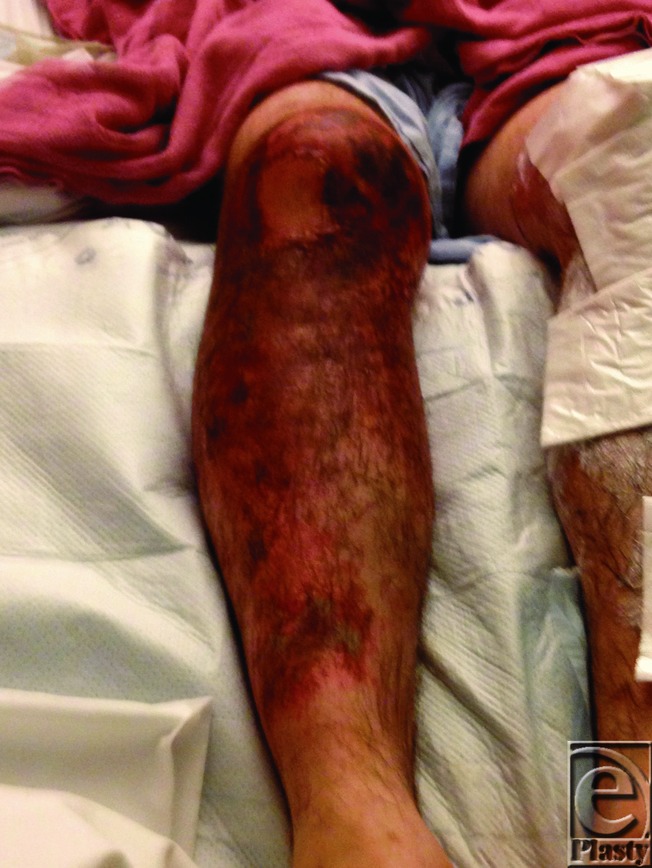


## DESCRIPTION

A 28-year-old man presented with bilateral anterior lower extremity burns after kneeling in wet cement for 3 hours while resurfacing the floor of his basement.

## QUESTIONS

1. **What are the clinical features?**
2. **How does cement cause injury?**
3. **Who is vulnerable to injury?**
4. **How are these injuries managed?**

## DISCUSSION

Typically, cement burns have an insidious onset with patients often initially observing only mild skin irritation. As such, the presentation is commonly late resulting in burns that are deep second or third degree. The injury is usually limited to the lower limbs, predominantly around the knee, foot, and ankle. The severity depends on the duration of contact between cement and skin.

The mechanism of injury is a combination of the effects of cement alkalinity and mechanical abrasion. The tricalcium silicate in cement reacts with water and sweat to release alkaline hydroxide (OH−) ions. The pH of the mixture can rise rapidly over 12 during this exothermic chemical reaction, 2Ca3SiO_5_ + 7H_2_O → 3CaO.2SiO_2_.4H_2_O + 3Ca(OH)_2_ + 173.6 kJ.^1^ Besides denaturing protein, alkalis saponify fat-producing liquefactive necrosis.

In the developed world, the majority of patients are either workers in the construction industry or do-it-yourself enthusiasts, commonly kneeling or standing in cement. In developing countries, however, most injuries are due to kiln explosions, often associated with an inhalation injury.^2^

Cement-soaked clothes should be removed and thorough irrigation of the burnt area with water should be performed until the pH approaches neutral. Traditionally, neutralization of alkaline burns is not recommended with the theoretical risk of deepening the injury by the resulting exothermic reaction. In an experimental study in a rodent model, however, improved healing was shown compared to the control group.^3^ Once a deep burn has been diagnosed, early excision and grafting is considered as the standard of care and it helps reduce recovery time and work absence, limits hypertrophic scarring, and provides durable skin.^4^
